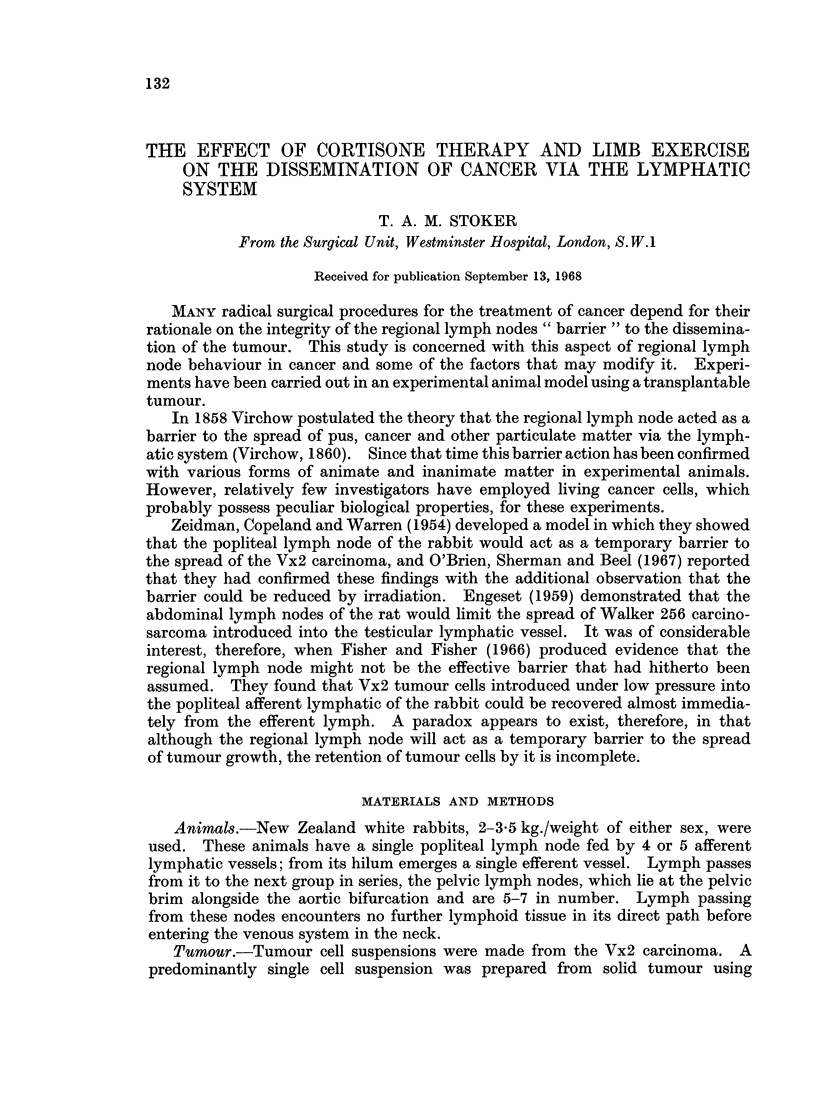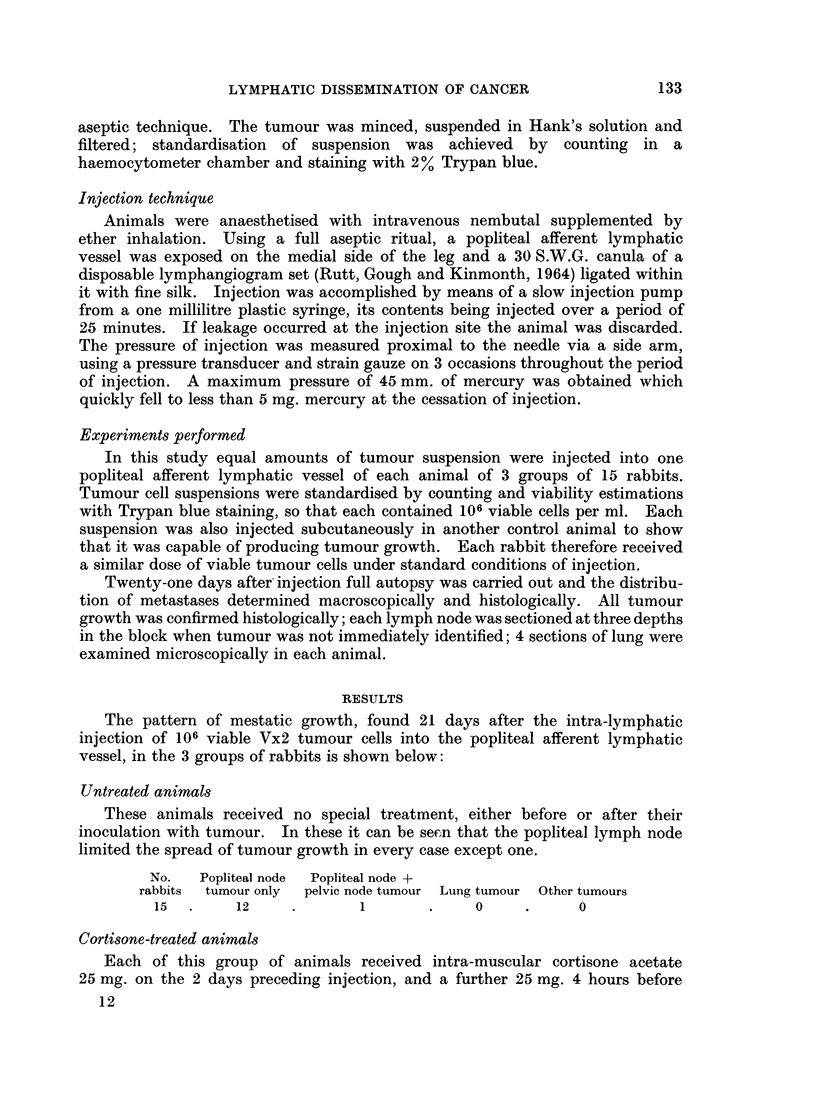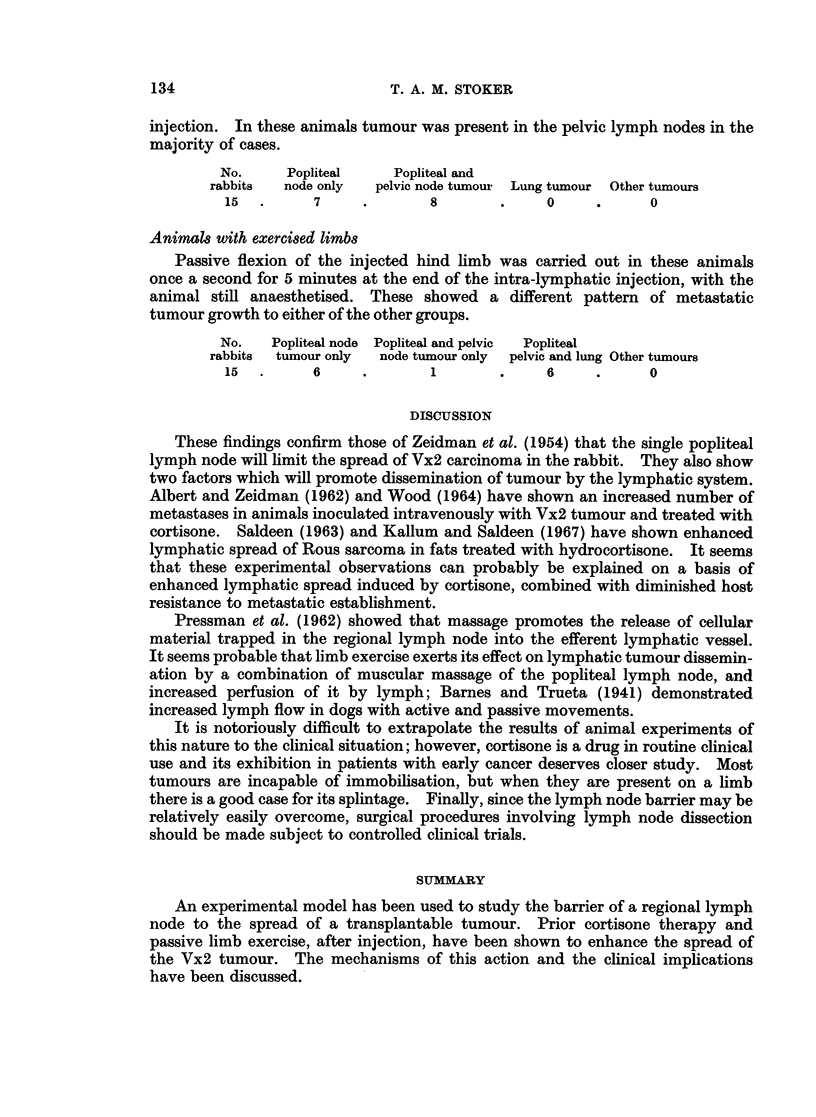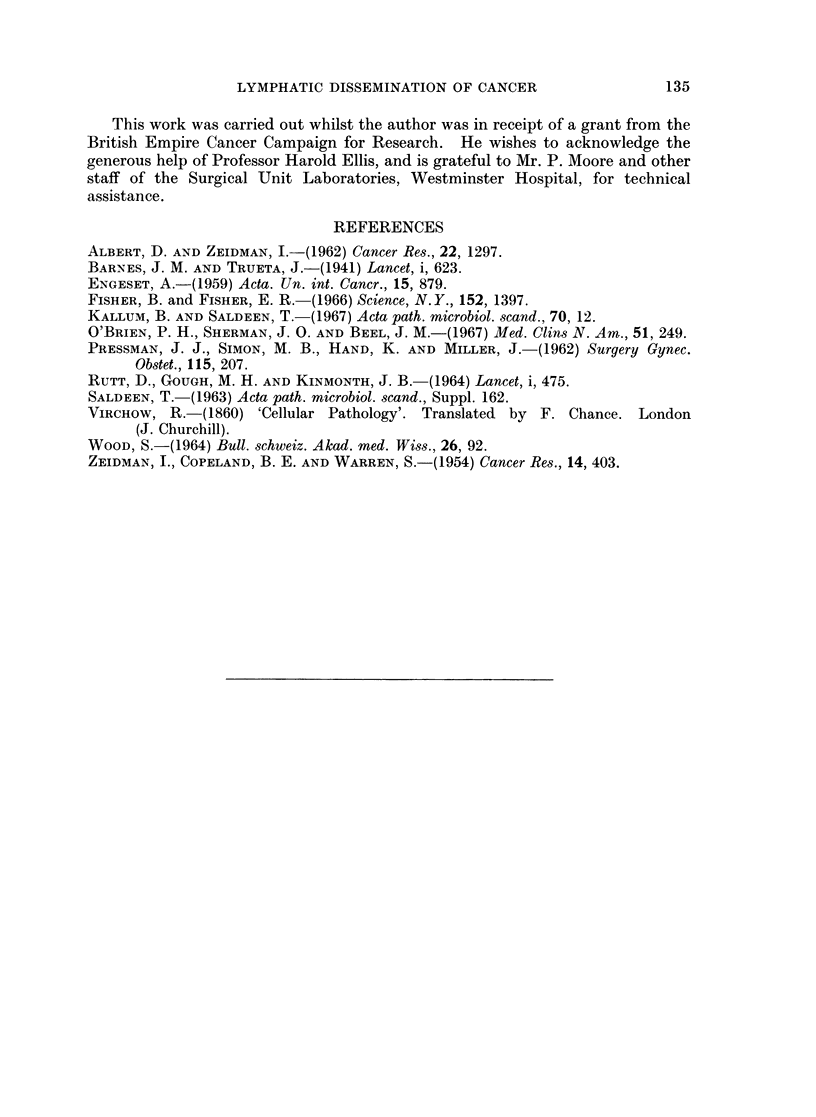# The effect of cortisone therapy and limb exercise on the dissemination of cancer via the lymphatic system.

**DOI:** 10.1038/bjc.1969.19

**Published:** 1969-03

**Authors:** T. A. Stoker


### THE EFFECT OF CORTISONE THERAPY AND LIMB EXERCISE ON THE DISSEMINATION OF CANCER VIA THE LYMPHATIC SYSTEM

T. A. M. STOKER

*From the Surgical Unit, Westminster Hospital, London, S.W.1*

Received for publication September 13, 1968

MANY radical surgical procedures for the treatment of cancer depend for their rationale on the integrity of the regional lymph nodes " barrier " to the dissemination of the tumour. This study is concerned with this aspect of regional lymph node behaviour in cancer and some of the factors that may modify it. Experiments have been carried out in an experimental animal model using a transplantable tumour.

In 1858 Virchow postulated the theory that the regional lymph node acted as a barrier to the spread of pus, cancer and other particulate matter via the lymphatic system (Virchow, 1860). Since that time this barrier action has been confirmed with various forms of animate and inanimate matter in experimental animals. However, relatively few investigators have employed living cancer cells, which probably possess peculiar biological properties, for these experiments.

Zeidman, Copeland and Warren (1954) developed a model in which they showed that the popliteal lymph node of the rabbit would act as a temporary barrier to the spread of the Vx2 carcinoma, and O'Brien, Sherman and Beel (1967) reported that they had confirmed these findings with the additional observation that the barrier could be reduced by irradiation. Engeset (1959) demonstrated that the abdominal lymph nodes of the rat would limit the spread of Walker 256 carcinosarcoma introduced into the testicular lymphatic vessel. It was of considerable interest, therefore, when Fisher and Fisher (1966) produced evidence that the regional lymph node might not be the effective barrier that had hitherto been assumed. They found that Vx2 tumour cells introduced under low pressure into the popliteal afferent lymphatic of the rabbit could be recovered almost immediately from the efferent lymph. A paradox appears to exist, therefore, in that although the regional lymph node will act as a temporary barrier to the spread of tumour growth, the retention of tumour cells by it is incomplete.

### MATERIALS AND METHODS

*Animals.*—New Zealand white rabbits, 2–3·5 kg./weight of either sex, were used. These animals have a single popliteal lymph node fed by 4 or 5 afferent lymphatic vessels; from its hilum emerges a single efferent vessel. Lymph passes from it to the next group in series, the pelvic lymph nodes, which lie at the pelvic brim alongside the aortic bifurcation and are 5–7 in number. Lymph passing from these nodes encounters no further lymphoid tissue in its direct path before entering the venous system in the neck.

*Tumour.*—Tumour cell suspensions were made from the Vx2 carcinoma. A predominantly single cell suspension was prepared from solid tumour using aseptic technique. The tumour was minced, suspended in Hank's solution and filtered; standardisation of suspension was achieved by counting in a haemocytometer chamber and staining with 2% Trypan blue.

*Injection technique*

Animals were anaesthetised with intravenous nembutal supplemented by ether inhalation. Using a full aseptic ritual, a popliteal afferent lymphatic vessel was exposed on the medial side of the leg and a 30 S.W.G. canula of a disposable lymphangiogram set (Rutt, Gough and Kinmonth, 1964) ligated within it with fine silk. Injection was accomplished by means of a slow injection pump from a one millilitre plastic syringe, its contents being injected over a period of 25 minutes. If leakage occurred at the injection site the animal was discarded. The pressure of injection was measured proximal to the needle via a side arm, using a pressure transducer and strain gauze on 3 occasions throughout the period of injection. A maximum pressure of 45 mm. of mercury was obtained which quickly fell to less than 5 mg. mercury at the cessation of injection.

*Experiments performed*

In this study equal amounts of tumour suspension were injected into one popliteal afferent lymphatic vessel of each animal of 3 groups of 15 rabbits. Tumour cell suspensions were standardised by counting and viability estimations with Trypan blue staining, so that each contained $10^6$ viable cells per ml. Each suspension was also injected subcutaneously in another control animal to show that it was capable of producing tumour growth. Each rabbit therefore received a similar dose of viable tumour cells under standard conditions of injection.

Twenty-one days after injection full autopsy was carried out and the distribution of metastases determined macroscopically and histologically. All tumour growth was confirmed histologically; each lymph node was sectioned at three depths in the block when tumour was not immediately identified; 4 sections of lung were examined microscopically in each animal.

### RESULTS

The pattern of mestatic growth, found 21 days after the intra-lymphatic injection of $10^6$ viable Vx2 tumour cells into the popliteal afferent lymphatic vessel, in the 3 groups of rabbits is shown below:

*Untreated animals*

These animals received no special treatment, either before or after their inoculation with tumour. In these it can be seen that the popliteal lymph node limited the spread of tumour growth in every case except one.

| No. rabbits | Popliteal node tumour only | Popliteal node + pelvic node tumour | Lung tumour | Other tumours |
|---|---|---|---|---|
| 15 | 12 | 1 | 0 | 0 |

*Cortisone-treated animals*

Each of this group of animals received intra-muscular cortisone acetate 25 mg. on the 2 days preceding injection, and a further 25 mg. 4 hours before injection. In these animals tumour was present in the pelvic lymph nodes in the majority of cases.

| No. rabbits | Popliteal node only | Popliteal and pelvic node tumour | Lung tumour | Other tumours |
|---|---|---|---|---|
| 15 . | 7 . | 8 . | 0 . | 0 |

*Animals with exercised limbs*

Passive flexion of the injected hind limb was carried out in these animals once a second for 5 minutes at the end of the intra-lymphatic injection, with the animal still anaesthetised. These showed a different pattern of metastatic tumour growth to either of the other groups.

| No. rabbits | Popliteal node tumour only | Popliteal and pelvic node tumour only | Popliteal pelvic and lung | Other tumours |
|---|---|---|---|---|
| 15 . | 6 . | 1 . | 6 . | 0 |

### DISCUSSION

These findings confirm those of Zeidman *et al.* (1954) that the single popliteal lymph node will limit the spread of Vx2 carcinoma in the rabbit. They also show two factors which will promote dissemination of tumour by the lymphatic system. Albert and Zeidman (1962) and Wood (1964) have shown an increased number of metastases in animals inoculated intravenously with Vx2 tumour and treated with cortisone. Saldeen (1963) and Kallum and Saldeen (1967) have shown enhanced lymphatic spread of Rous sarcoma in fats treated with hydrocortisone. It seems that these experimental observations can probably be explained on a basis of enhanced lymphatic spread induced by cortisone, combined with diminished host resistance to metastatic establishment.

Pressman *et al.* (1962) showed that massage promotes the release of cellular material trapped in the regional lymph node into the efferent lymphatic vessel. It seems probable that limb exercise exerts its effect on lymphatic tumour dissemination by a combination of muscular massage of the popliteal lymph node, and increased perfusion of it by lymph; Barnes and Trueta (1941) demonstrated increased lymph flow in dogs with active and passive movements.

It is notoriously difficult to extrapolate the results of animal experiments of this nature to the clinical situation; however, cortisone is a drug in routine clinical use and its exhibition in patients with early cancer deserves closer study. Most tumours are incapable of immobilisation, but when they are present on a limb there is a good case for its splintage. Finally, since the lymph node barrier may be relatively easily overcome, surgical procedures involving lymph node dissection should be made subject to controlled clinical trials.

### SUMMARY

An experimental model has been used to study the barrier of a regional lymph node to the spread of a transplantable tumour. Prior cortisone therapy and passive limb exercise, after injection, have been shown to enhance the spread of the Vx2 tumour. The mechanisms of this action and the clinical implications have been discussed.

This work was carried out whilst the author was in receipt of a grant from the British Empire Cancer Campaign for Research. He wishes to acknowledge the generous help of Professor Harold Ellis, and is grateful to Mr. P. Moore and other staff of the Surgical Unit Laboratories, Westminster Hospital, for technical assistance.

### REFERENCES

ALBERT, D. AND ZEIDMAN, I.—(1962) *Cancer Res.*, **22**, 1297.
BARNES, J. M. AND TRUETA, J.—(1941) *Lancet*, i, 623.
ENGESET, A.—(1959) *Acta. Un. int. Cancr.*, **15**, 879.
FISHER, B. and FISHER, E. R.—(1966) *Science, N.Y.*, **152**, 1397.
KALLUM, B. AND SALDEEN, T.—(1967) *Acta path. microbiol. scand.*, **70**, 12.
O'BRIEN, P. H., SHERMAN, J. O. AND BEEL, J. M.—(1967) *Med. Clins N. Am.*, **51**, 249.
PRESSMAN, J. J., SIMON, M. B., HAND, K. AND MILLER, J.—(1962) *Surgery Gynec. Obstet.*, **115**, 207.
RUTT, D., GOUGH, M. H. AND KINMONTH, J. B.—(1964) *Lancet*, i, 475.
SALDEEN, T.—(1963) *Acta path. microbiol. scand.*, Suppl. 162.
VIRCHOW, R.—(1860) 'Cellular Pathology'. Translated by F. Chance. London (J. Churchill).
WOOD, S.—(1964) *Bull. schweiz. Akad. med. Wiss.*, **26**, 92.
ZEIDMAN, I., COPELAND, B. E. AND WARREN, S.—(1954) *Cancer Res.*, **14**, 403.